# A grounded theory of how social support influences physical activity in adolescent girls

**DOI:** 10.1080/17482631.2018.1435099

**Published:** 2018-02-06

**Authors:** Yvonne Laird, Samantha Fawkner, Ailsa Niven

**Affiliations:** a Scottish Collaboration for Public Health Research and Policy (SCPHRP), University of Edinburgh, Edinburgh, UK; b The Physical Activity for Health Research Centre (PAHRC), University of Edinburgh, Edinburgh, UK; c Institute of Sport, Physical Education and Health Sciences, University of Edinburgh, Edinburgh, UK

**Keywords:** Co-participation, encouragement, family, friends, modelling, parents

## Abstract

**Purpose**: Adolescent girls are not sufficiently active to achieve health benefits. Social support from friends and family has been positively associated with physical activity in adolescent girls; however it is unclear how social support influences physical activity behaviour. This study aimed to develop a grounded theory of how social support influences physical activity in adolescent girls. **Methods:** A qualitative, constructivist grounded theory approach was adopted. Individual interviews explored adolescent girls’ perspectives of how significant others’ influenced their physical activity through providing social support, and through modelling physical activity. **Results:** Participants perceived social support to influence physical activity behaviour through performance improvements, self-efficacy, enjoyment, motivation and by enabling physical activity. Improvements in performance and self-efficacy were also linked to motivation to be active. Girls perceived modelling to influence behaviour through providing opportunities for them to be physically active, and by inspiring them to be active. **Conclusion:** The grounded theory outlines adolescent girls’ perceptions of how significant others influence their physical activity and provides a framework for future research examining the role of social support on physical activity.

## Introduction

Physical inactivity is a leading risk factor for the development of non-communicable diseases, and consequently, is a major global public health concern (Ding et al., 2016). The health benefits of regular physical activity (PA) for young people are well-documented (Janssen & LeBlanc, 2010; Poitras et al., 2016), yet an estimated 80% of adolescents aged 13–15 years worldwide fail to reach minimum PA guidelines of 60 minutes of moderate-to-vigorous PA (MVPA) per day (Hallal et al., 2012). Girls are consistently identified as less active than boys (Currie et al., 2015; Hallal et al., 2012), with an estimated 95% of adolescent girls aged 13–15 years worldwide failing to reach minimum PA guidelines (Hallal et al., 2012). As such, adolescent girls have been identified as a priority group for increasing PA levels (The Physical Activity for Health Alliance, 2010). However, interventions aimed at increasing PA in adolescent girls have had limited success (Pearson, Braithwaite, & Biddle, 2015). There is some indication of gender differences between the factors related to PA in adolescents (Telford, Telford, Olive, Cochrane, & Davey, 2016), and as such, interventions may need to be tailored to be effective for boys or girls. A better understanding of the factors associated with PA in adolescent girls specifically may inform more effective intervention design for this population (Sallis, Owen, & Fotheringham, 2000).

Systematic reviews have synthesized evidence on correlates associated with PA in adolescent girls (Biddle, Atkin, Cavill, & Foster, 2011; Standiford, 2013). These reviews have identified personal and demographic, psychological, environmental and social correlates to be consistently associated with adolescent girls’ PA behaviour. Social networks, or specific sets of linkages between people (Mitchell, 1969), can influence PA through various mechanisms. One mechanism that may be particularly important for adolescent girls is social support. Social support has been defined as resources provided from interactions with significant others (e.g., parents, friends) within a social network (Langford, Bowsher, Maloney, & Lillis, 1997; Sheridan & Radmacher, 1992). Social support can be emotional (e.g., encouragement, praise), instrumental (e.g., logistic support), informational (e.g., instruction), or co-participation (e.g., engaging in PA with child) (Heaney & Israel, 2008; Yao & Rhodes, 2015). Additionally, social support can be *perceived* (e.g., the support a person thinks that they get) or *received* (e.g., the support a provider thinks that they give). Perceived versus received support may be important for PA behaviour. For example, a person may believe they are supporting a child to be active but if this is not perceived by the child then the influence on PA behaviour may vary. Within the wider literature, perceived social support has been found to be more predictive of positive health outcomes (e.g., Holt-Lunstad, Smith, & Layton, 2010; McDowell & Serovich, 2007; Uchino, Bowen, Carlisle, & Birmingham, 2012), although no evidence of differential effects between perceived versus received support were identified for social support on girls’ PA behaviour (Laird, Fawkner, Kelly, McNamee, & Niven, 2016).

Although several reviews have considered the role of social networks, in particular social support, on PA in children and adolescents (e.g., Beets, Cardinal, & Alderman, 2010; Edwardson & Gorely, 2010; Yao & Rhodes, 2015), only one focused specifically on adolescent girls (Laird et al., 2016). This review identified significant positive associations between social support from friends and family and adolescent girls’ PA. The review also highlighted the complexity of the relationship between social support and PA, with numerous types and providers of support identified, and varying effect sizes for these associations with PA identified between studies. This suggests a need to unpick this relationship to better understand the complexity of the relationship between social support and PA in adolescent girls.

Given that positive associations between social support and PA have been identified, PA interventions have attempted to increase young peoples’ perceptions of social support. Intervention strategies have included promoting positive communication, teamwork, engaging families in interventions, and encouragement (Dunton, Schneider, & Cooper, 2007; Lubans & Sylva, 2009). However, these strategies have had limited success at increasing perceptions of support (van Stralen et al., 2011).

A better understanding of *how* social support influences PA behaviour could inform more effective PA intervention design. To date, only limited research has investigated the ways in which social support influences PA behaviour. Cross-sectional studies have consistently shown that self-efficacy is a mediating variable between social support and PA (Motl, Dishman, Saunders, Dowda, & Pate, 2007; Peterson, Lawman, Wilson, Fairchild, & Van Horn, 2013; Wing, Bélanger, & Brunet, 2016). That is, social support may enhance an individual’s self-efficacy to overcome barriers to PA, and this enhanced self-efficacy is associated with greater PA. Other studies have highlighted that lack of time, lack of interest and enjoyment may also mediate the relationship between social support and PA (Shen et al., 2016; Verloigne et al., 2014; Wing et al., 2016), but these findings are based on only three studies. This suggests that a lack of social support may lead to young people being uninterested in PA and perceive barriers, such as lack of time, to PA. This perceived lack of interest and time is associated with low levels of PA. Conversely, social support may enhance young peoples’ enjoyment of PA, and this enhanced enjoyment is linked with greater participation in PA. Two qualitative studies have considered how social support influences PA behaviour. Weiss and colleagues (1996) found some evidence to suggest that friends influence perceptions of self-esteem, enjoyment and performance in sports. A focus group study by Jago and colleagues (2009) found that support from friends encouraged young people to try new physical activities, and co-participation with friends was linked with enjoyment of PA. The focus of these papers was not on understanding how support influences PA, as such other potential mechanisms of how support influences PA may not have been explored. Whilst these findings give some indication of the possible ways in which social support influences PA, it is important to note that the majority of these studies considered boys and girls together. Considering potential gender differences, a specific focus on how social support influences adolescent girls’ PA may be important to inform targeted PA interventions for adolescent girls. As there has been limited prior research, it is also possible that there are other pathways through which social support influences PA.

Investigating girls’ perspectives and experiences of social support provided by significant others for PA behaviour would improve our understanding of how social support influences PA behaviour, which in turn could inform the development of PA intervention strategies aimed at engaging inactive girls in PA. This study aimed to advance current research on the influence of social support on PA in adolescent girls by qualitatively investigating the mechanisms through which adolescent girls perceive social support to influence their PA behaviour. In particular, this study aimed to: (1) identify how girls perceive different sources and types of support to influence their PA behaviour, and (2) develop a grounded theory of how social support influences PA in adolescent girls to inform intervention design.

## Method

Constructivist grounded theory provides a methodology to develop a rich theoretical understanding of adolescent girls’ perceptions and experiences of how social support influences their participation in PA. In-depth individual interviews could provide a deeper understanding of the pathways through which social support influences PA, allowing participants to describe their experiences, insights and the context of their experiences. A constructivist grounded theory approach outlined by Charmaz (2006, 2014), guided all stages of the research process from study design to data analysis and the resulting grounded theory. The first author conducted all of the interviews and carried out the analysis with input from the co-authors. All authors had prior experience of qualitative research with adolescent girls exploring PA. This project formed part of the first authors’ doctoral research exploring the role of social support on PA in adolescent girls. Whilst the study authors had prior knowledge of the topic area at the time of conducting the study, they kept open minds about the ways in which social support might influence PA.

### Participants

As the study aims relate to *perceived* social support, we sampled adolescent girls to investigate their perspectives of how social support influences their participation in PA. Girls in year 3 of secondary school aged 13–15 years from two purposefully sampled schools in an urban city in the UK were invited to participate. Schools were selected because their catchment areas covered multiple levels of deprivation. Classes within these schools were chosen by teachers based on potential to identify the target population. All girls from a total of four classes within the two schools completed a single-item questionnaire to estimate their PA levels (Prochaska, Sallis, & Long, 2001) and were invited to participate in the study. The PA questionnaire asked participants to circle the number of days in the previous week that they were physically active for 60 minutes at a moderate-to-vigorous intensity, in line with UK PA guidelines (Department of Health, 2011). The PA questionnaire was used to guide selection of participants. We aimed to recruit physically active adolescent girls as we expected active girls would be more likely to perceive support for PA than inactive girls, and hence, more likely to be able to describe how social support influences their behaviour. In this study, we considered PA as being any bodily movement produced by the skeletal muscles that results in energy expenditure (Caspersen, Powell, & Christenson, 1985). All forms of PA were considered within the context of providing social support, for example active travel, organized sports involvement, leisure activities and walking.

### Procedures

All study procedures were approved by the local institutional ethics committee and by relevant local authorities. Three schools were invited to participate, of which two agreed to take part. Potential participants were given at least 1 week to read and consider a study information letter and decide if they would like to participate, as well as the opportunity to ask the first author any questions about the participating in the research. Participants and their parents completed informed consent. All personal data collected, including signed consent forms and screening questionnaires, were stored securely in a locked filing cabinet within the university. Following recruitment, participants were selected to take part in an in-depth individual interview with the first author. Interviews were conducted in school at a time agreed with teachers and participants. The initial sample involved selecting the most active girls who were reaching or close to reaching PA guidelines, based on responses to the single-item PA questionnaire (Prochaska et al., 2001). Based on early analysis of the data, we decided not to select participants based on their responses to the single-item PA questionnaire. It became clear through the girls’ verbal descriptions of their activity levels during the interviews that the single-item PA questionnaire underestimated activity levels. Instead, we consulted teachers to help identify suitable participants, which also assisted with following up on the ongoing analysis, emergent findings and theoretical sampling.

### Instrument

A semi-structured interview guide was developed containing questions designed to elicit open responses with accompanying prompts and probes. Questions explored: (1) participants’ PA involvement, (2) the PA involvement of the participants’ friends, family and other network members, (3) social support for PA provided to participants’ from those in their social networks, and (4) how the girls perceived this social support to influence their PA behaviour. Participants and their network members’ involvement in PA was considered within the broad definition of PA outlined previously. Participants were prompted to describe all forms of PA they and their network members participated in, for example organized sports, walking, active travel and leisure activities, as well as the duration and frequency of this involvement. Early questions were designed to act as icebreakers, to ease participants into the interview. The interview guide was piloted prior to data collection in a role play scenario with a physically active female adult to give the researcher the opportunity to practice interviewing and to receive feedback on the topic guide and interviewing style. Following this, minor modifications were made to improve the clarity and flow of questions in the interview guide. Piloting the interview guide with an adolescent girl would have provided a better indication of the suitability of the questions and structure for adolescent girls, however, as data collection was an iterative process, the suitability and effectiveness of the interview schedule was considered after each interview and was adapted to follow-up on emerging themes and concepts.

### Data analysis

Interviews were audio-recorded using a digital voice recorder (Olympus VN-713PC, Toyko) then transcribed verbatim, excluding any information that could identify the participant, yielding 176 pages of single-spaced text. Interview transcripts were uploaded to NVivo10 software (QSR International Pty Ltd, vs. 10, 2012), where data were stored and analyzed. Each interview was conducted, transcribed, and analyzed before the next interview was carried out. This iterative process informed the sampling strategy, developments to the interview schedule, and the evolving themes that emerged from the data.

### Coding data

Coding was carried out in two stages following procedures outlined by Charmaz (2014) and facilitated using the “nodes” function in NVivo10. The first stage involved initial coding, whereby codes were closely linked to the data. This process allowed the first author to develop a broad understanding of the data. As data collection and analysis continued, initial codes were refined. The second stage, focused coding, involved a more analytical approach. Focused coding refined the initial codes through highlighting important aspects of the analysis. This included identifying patterns in the data and identifying initial codes that categorized the data. Some initial codes were grouped together and recoded.

Constant comparison methods (Glaser & Straus, 1967) were used at all stages of analysis. Comparisons began when analysing the initial interview transcript by identifying similarities and differences. Data were then compared within and between interviews. As data collection and analysis progressed, codes and data from the final interviews were compared with codes and data from the early interviews to check that later interpretations of the data were relevant and applicable to early data and analysis.

Thoughts, ideas, and interpretations of the data were recorded by memo-writing. Memos were written following each interview to reflect on the interview and the analysis. Memo-writing helped to develop ideas, assisted with the constant comparison process and helped to form the grounded theory used to represent the data. Memo-writing was also used to help the first author reflect on the data collection process. For example, noting strengths and areas for improvement for subsequent interviews. A methodological journal was also used to promote additional reflexivity. Thoughts, ideas and conceptualizations of the data were noted and linkages were explored. The methodological journal also guided coding and the emerging grounded theory that was used to represent the data.

In recognition of the complexity of ensuring and demonstrating the results truly reached the point of saturation, data collection continued until the authors were satisfied that the data collected were sufficiently rich and answered the research questions (O’Reilly & Parker, 2012). By adopting this viewpoint, we acknowledge the potential for further research to expand, develop or modify the resultant grounded theory, and recognize that our sample may not be representative of all adolescent girls or fully explanatory to all domains of PA. On completion of coding all transcripts, memos and methodological journal entries were re-read and all coding in NVivo was checked to ensure consistency.

### Development of the grounded theory

The coding, memos and methodological journal were used to develop the grounded theory. The findings were compared with previous research to inform the final model, which is a common approach in constructivist grounded theory (Sabiston, McDonough, & Crocker, 2007). The final grounded theory was presented to a sub-sample of the participants (*n* = 6). The sub-sample was a convenience sample selected by the teachers. Each part of the theory was outlined and discussed with the participants in a group setting to check participants’ understanding, and to establish whether it accurately represented their thoughts and experiences. Participants were given the opportunity to discuss and suggest modifications to the theory. The sub-sample of participants confirmed that the final version of the grounded theory represented their thoughts and experiences.

### Trustworthiness

Criteria for assessing the quality of qualitative studies are often termed as trustworthiness (Elo et al., 2014). Trustworthiness can be assessed at each stage of a research process, including data collection, interpretation, organization and reporting of results (Elo et al., 2014). Within grounded theory, quality is achieved by immersing oneself in the process and closely following grounded theory methodology. Weed (2009) suggests eight core elements that are necessary to meet quality conditions for grounded theory including: an iterative process; theoretical sampling; theoretical sensitivity; codes, memos and concepts; constant comparison; theoretical saturation; fit, work, relevance and modifiability; and substantive theory. We have already outlined how each of these criteria were met, except for fit, work, relevance and modifiability and substantive theory. Fit refers to how closely the theory represents the data and phenomenon of interest. Fit is ensured by constant comparison and theoretical saturation. A theory is thought to “work” if it provides an explanation for the problem of which it represents. The “relevance” of a theory is how closely a theory represents the people it is based on, and “modifiability” means that a theory can be further developed. Substantive theory means that a grounded theory is specific to a particular area, and not universally applicable. It is thought that several substantive grounded theories can be joined to create a more generally applicable grounded theory. The reader is encouraged to make their own judgements on the quality of this grounded theory study and make assessments of its “fit, work, relevance, and modifiability.”

## Results

### Participant characteristics

Participants were 18 girls aged between 13 and 15 years. Participants discussed being involved in a range of physical activities including organized sports, active commuting, Physical Education (PE) classes, and leisure activities. The majority of girls (*n* = 16) were involved in at least one organized sport including hockey (*n* = 7), basketball (*n* = 4), football (*n* = 5), dancing (*n* = 3), taekwondo (*n* = 1), karate (*n* = 2) and tennis (*n* = 1). Six girls were involved in more than one organized sport. The majority of girls (*n* = 16) walked to and/or from school and all of the girls had chosen to do PE as an academic subject, which meant they were doing PE up to 5 times per week. The two girls who were not involved in organized sports regularly walked or jogged for leisure, and walked to and from school every day as well as taking part in PE.

### Grounded theory of how social support influences physical activity in adolescent girls

**Figure 1.**
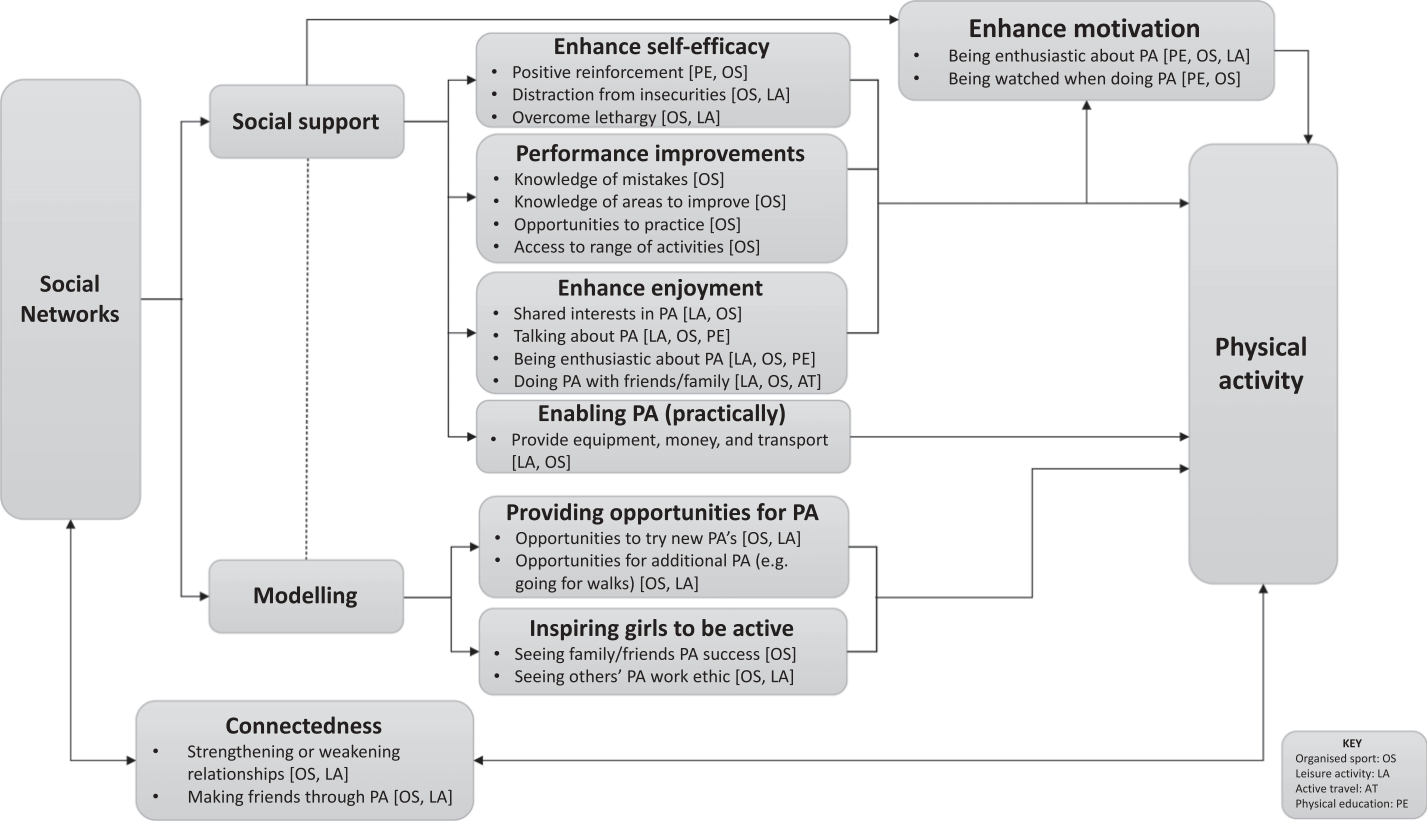
Grounded theory of how social support and modelling influences physical activity in adolescent girls.

The final grounded theory, presented in Figure 1, represents the ways in which participants perceived their social networks to influence different domains of their PA behaviour, including organized sport participation, leisure activities, active transport and PE. Social networks (the core-category) were proposed to influence PA through three categories: provision of social support, modelling and connectedness. Modelling emerged as important for many of the participants and closely linked to provision of social support, and as such, is included in the grounded theory. Additionally, connectedness (strength and number of relationships with friends and family) was found to have an important role in this grounded theory as a link emerged between involvement in PA, connectedness, and capacity for networks to provide support. The grounded theory outlines the sub-categories by which these categories might influence PA behaviour. The sub-categories and categories emerged from coding in NVivo. For example, the code “friends help pacing” depicted how being active with friends provided opportunities for practice (sub-category) and knowledge of areas to improve (sub-category), which could lead to performance improvements (category). The following sections describe each construct and pathways in more detail and provide illustrative quotes. Participant names have been replaced throughout with random, computer-generated pseudonyms.

#### Social support

Social support was found to influence PA through five sub-categories: enhancing self-efficacy, performance improvements, enhancing enjoyment, enhancing motivation and enabling PA (practically). The following section outlines these sub-categories.

##### By enhancing self-efficacy

Social support was found to enhance both task and barrier self-efficacy. Task self-efficacy is a persons’ confidence in their ability to be physically active (McAuley & Mihalko, 1998). Participants emphasized how social support contributed to PA task self-efficacy, particularly through receiving reinforcement and praise for physical activities. The following quote emphasizes how praise and positive reinforcement increased Maria’s self-efficacy and highlights how self-efficacy can lead to sustained engagement in organized sport.

Maria … “there’s been a game I can think of where em the teacher that was watching kind of took me aside afterwards and spoke to me about my confidence on the pitch… she just reminded me that I had every right to be where I was and that I, I shouldn’t be lacking in confidence… it made me feel a lot more confident about just turning up for the games and feeling that I mean, sometimes, feeling that your team might need you to do something and that made me feel really good, so.”

There was some indication that in order to feel more confident about their PA abilities the girls had to believe the provider genuinely believed in their abilities.

Karen “… well they obviously think that, if they’re being this tough on me [referring to the various ways her parents support her] they obviously think that I have potential, so they know, well they have faith in me to be good at the sport so it pushes me on a wee bit more.”

Task self-efficacy also enhanced PA through motivation. As demonstrated by the following quote, PA self-efficacy resulting from social support contributed to participants’ determination and motivation in organized sport.

Stacey “I like getting compliments about it because I don’t really think I’m that good but, like, when I get compliments about it, it just makes my confidence grow a bit higher with it… I think—right I’m good at this, I need to try harder to get even better at it. So I’m trying even harder than I was before to achieve something better.”

The girls also described how social support helped them to overcome barriers to being physically active, or barrier self-efficacy. For example, one girl described how feeling self-conscious was a barrier to going jogging by herself but having a friend helped her feel more confident.

Rebecca “… when you go out sometimes you feel a bit awkward being on your own, or I feel awkward being on my own in case, like, people are looking at me in a weird way. But if you’re with someone else you feel a bit more, em, confident.”

Feeling self-conscious about being active was identified by several of the girls as a barrier to starting new organized sports and leisure activities. However, Stacey described how having friends to be active with helps to overcome barriers to trying new activities.

Stacey “… as you get older, like in this kind of phase, you’d want people there because you’re quite insecure and stuff like that. Like, if people are laughing in a group you’d think they were laughing at me because you’re not doing it right or because you’ll need more attention because you’ve just joined. When you’re with friends you just laugh about it, ‘oh, I done that wrong, big deal!’, but when you’re by yourself you’re more insecure.”

Lethargy was also described by some of the girls as a barrier to being active. Girls discussed how encouragement from family and friends helped them to overcome feelings of lethargy to take part in organized sports or leisure activities.

Stacey “… if I’m in my room and they’re like, ‘come out’, and I’m like, ‘ugh, I can’t be bothered!’, and they’ll be like, ‘come on, just come out! You can laze about every day!’ … Like, I’ll go out and I’ll end up having a really good time, like, I’m glad that I’ve went out.”

##### Performance improvements

Participants linked specific forms of social support with performance improvements in organized sports. These improvements in performance were linked with continuation in organized sports. Girls described how significant others identified areas where they could improve and provided them with advice or instruction. To demonstrate this, Karen’s parents identify areas that she can work on in training through watching her basketball matches:

Karen “… well I’ve been playing basketball for four or five years now so yeah the more that I’ve done they’ve got quite a good knowledge of it now as well so and they can see more, they can see more than me of how well I do on the court than I can see of myself if that makes sense.”

Researcher “… so having someone watch you helps pick up faults or?”

Karen “Yeah, pick up faults or if I didn’t have the right attitude or I didn’t, if I kept doing the same mistake over and over again or if I let someone steal the ball off me too many times and my head went down. Something like that. They can see all that more than I realize it so it does help. We know what I need to improve on or what I need to do for the next game.”

Many of the girls talked about how they valued this support. In particular, girls discussed how personal feedback from parents or friends was beneficial by helping them to identify areas that they could improve. One girl noted that feedback from coaches was normally less personal, so also receiving feedback from her parents was particularly helpful to her.

Social support and social networks also presented opportunities for the girls to improve their PA skills through skills practice and access to a broader range of activities. For example, Sarah said she occasionally hired a basketball court with friends to practice. She noted how being physically active with friends helps to improve her basketball.

Sarah “… you can do lots of different stuff with a second person there whereas if you’re on your own you can really only do ball handling stuff, you can only do shooting, whereas when you’re with another person you can go one v one, you can go eh ball handling with a defender there, you can get passing in. It’s generally better having that second person there.”

A link was also evident between performance improvements, motivation and sustained engagement in PA. Maria spoke about how noticing improvements in her performance in sport was motivating.

Maria “… when you see an improvement that’s a really, really, a nice feeling to have… that’s really motivating.”

##### By enhancing motivation

In addition to being motivated through self-efficacy and performance improvements, motivation was a key outcome of provision of social support on its own, and was described by participants as directly related to their participation in PE, organized sports and leisure activities. Girls discussed various ways in which support from friends, families, teachers and coaches motivated them. For example, girls discussed how having a shared interest in physical activities with friends was motivational.

Maria “… we all do a lot of hockey training together and I suppose just talking about trainers and running leggings and things, it, it helps em and just, I sometimes think that if I didn’t do the sport with them I would have a lot less in common with my friends so it kind of encourages you and motivates you.”

In a PE setting, supportive teachers facilitated participation in lessons through enthusiasm, providing encouragement, taking about physical activities, watching the girls during lessons and praising and providing feedback on physical activities. Rebecca discussed how teachers watching her during PE makes her want to put in more effort:

Rebecca ‘I think it motivates me to like work harder “cos especially like when you know they’re watching so you’re like, ‘Oh, I need to try’, so that, like, helps.”

Similarly, support from friends, family or teachers motivated participation in physical activities outside of PE. For example, Karen talked about how support from her friends can motivate her during training sessions to keep going:

Karen “… if I’m feeling like I can’t really be bothered and I’m down at training they can try and bring me back up and be enthusiastic and stuff… it pushes me to play a bit better.”

Participants described how encouragement and support more generally motivated them to be active. To demonstrate this, Karen discussed how a supportive team environment motivated her in physical activities.

Karen “Just, em, more determined to get better and, I don’t know, just kinda like with my mum and dad, you know, coming and telling me what to do as well it just pushes me on… so them doing more exercise, we do more exercise together, we get better as a team and stuff.”

##### By enhancing enjoyment

Friends, family, teachers and coaches were integral to making all domains of PA more enjoyable, which as a result, contributed to the girls’ engagement in PA. Teachers and coaches showed enthusiasm during training or lessons, engaged in positive conversations about physical activities with the girls, and went out of their way to organize additional physical activities. The girls discussed how these actions contributed to their enjoyment of physical activities, through creating a positive environment in which to be active. Participants frequently talked about how teachers and coaches made PA fun.

Annie “He’s, like, he’s really good. I really like him. He’s funny and he’s, just, like takes the mickey out of everybody. He’s just really funny and he’s a good coach… I think if you don’t have a good coach then you’re not as likely to listen and you’re not exactly, you’re not as likely to take it all in as somebody who has a bit of fun rather than just being boring and talking all the time.”

Having friends or family to be physically active with also made activities more enjoyable and contributed to positive experiences in PA. Some of the girls talked about how walking to school was better with friends than walking alone because they enjoyed having someone to talk to. Not having friends to walk with was identified as a barrier to walking to school by one girl. Similarly, another girl discussed how going to an exercise class with a friend made it fun but she did not think she would go alone because they were the only young people who attended the class.

Margaret “… with the classes at the gym, because it’s like mostly adults, we’re, like, the only kids. I don’t think I’d really, I don’t really want to go on my own… I think doing classes with a friend is more fun.”

Girls also discussed doing physical activities with friends in their free time, such as playing a game of football after school or playing basketball over breaks and lunches. The girls identified being active with friends as fun, and as such, many of the girls looked for opportunities to engage in PA with friends. Rose talked about how most days after school she plays football in the park with her friends:

Rose “They just go out and we all play a big game [of football] … Just to get out and have some fun.”

It addition, enjoyment was the most frequently mentioned reason for continuation of organized physical activities. Whilst it is likely that there are many contributing factors related to girls’ enjoyment of PA, having friends involved and a supportive environment in which to be active was a common reason why girls continued physical activities in this study.

Lori “I like hockey because I play it with my friends and because I’ve played it for ages so I’m ok. I’m quite good at it.”

##### Enabling PA (practically)

One of the primary ways girls identified receiving support, particularly from their parents, was through logistic forms of support such as providing transport to physical activities or paying for sports club or gym memberships and kit. Although some of the girls reported contributing their own money to fund their activities or that they walked or got the bus to training and did not rely on their parents for transportation, most of the girls relied on their parents for logistic support for physical activities and this logistic support directly enabled them to be active. Several of the girls noted that they would not be able to do the activities they do without this support from their parents.

Diana ‘… I get a lot of lifts but I don’t think if they couldn’t kind of give me lifts places and take me there then, like, I couldn’t do it all “cos it would be so much. I couldn’t get there in time for stuff, so.”

Susan ‘… “cos they pay for it, like, I wouldn’t be able to, like, pay for it.”

#### Modelling

Modelling is typically measured in terms of associations between PA levels of the provider and child (Laird et al., 2016). For the purpose of this study, we also considered modelling to include the providers’ interest and value in PA, as well as their PA levels. Modelling was viewed as conceptually unique from social support in this study, yet also linked to participants’ PA behaviour and the social support that was provided to participants. Modelling was found to influence PA through two sub-categories: providing opportunities to be active, and inspiring girls to be active.

##### Providing opportunities to be physically active

Participants frequently discussed how their friends and family, or other network members, were one of the reasons they first started an organized PA or sport. Parents introduced physical activities to their daughters at a young age or suggested physical activities for them to try, and having friends or family involved in physical activities presented opportunities for the girls to try it themselves.

Maria “… my friend was doing it [*hockey*] and she said, ‘Oh, you should come along’, so I came along and then I liked it and I had other people who I was friendly with there. It was fun and exercise so I just continued to go.”

Having friends or family members who were active themselves also presented opportunities to participate in leisure activities (e.g., walking, recreational swimming) through network members inviting girls along to activities that they were doing. For example, one participant discussed how her parents enjoy going for walks and how it was something that they enjoyed doing as a family:

Diana “… my mum and dad quite like going out for walks and stuff and they like to go out for coffee and stuff as well so normally we do stuff like that as a family as well.”

##### Inspiring girls to be active

Having members of social networks model physical activities and perform well in organized physical activities inspired some of the girls to take up physical activities or motivated them to continue activities they were already involved with. Stacey discussed how her brother’s success in boxing inspired her try boxing.

Stacey “… when my brother was winning, like, all his matches and stuff I started boxing because I saw, like, all his trophies and then the big smile and everyone being like, ‘aw, I’m so proud of you’, and stuff.”

Coaches were also a source of inspiration for some of the girls, particularly through their previous successes and through their knowledge and ability in physical activities. This inspired some of the girls to want to achieve similar levels of sporting success.

Paula “… sometimes we get to see them in, like, coach versus the seniors’ games. It’s to see them play the seniors, so it’s good to see them play because, like, they’re really good and it makes you think, ‘oh, I’d like to be able to play like that’.”

Girls also valued their coaches’ work ethic and performance achievements.

Paula “… they do inspire you because they, they like worked really hard and they’ve, a lot of them have, like, have done really well with hockey in the past so a lot of them have a lot of knowledge about it and its good for them to show us, like, what they know.”

#### Connectedness

Connectedness was found to influence PA through two sub-categories: strengthening or weakening relationships, and making friends through PA. Participating in PA was also found to enhance social networks by strengthening current relationships and by adding to social networks. A bidirectional relationship was evident (as shown in Figure 1), as girls described how PA could also negatively influence relationships. Many of the girls discussed making friends as a result of taking part in organized physical activities and they described how physical activities strengthened their current relationships.

Maria “My other grandparents… they like to have weekly sports roundup, em, and we always text on the Saturday and Sunday results and… everyone when I think about it, everyone seems to find out one way or another and everyone’s just involved.”

Two girls also spoke about how having family members’ interested and involved contributed to their enjoyment in physical activities. When asked why basketball was her favourite sport, Sophie replied: “…cos all my family do it… my mum plays it, my auntie plays it, and some of my cousins play it as well.”

Participating in PA also had a potentially negative impact in some cases on relationships and in participation in PA, suggesting a possible circular relationship between PA and connectedness (as illustrated in Figure 1 with bidirectional arrow). In instances where friends or family did not have a shared interest in PA this could impact negatively on the relationship or on the girls’ participation in PA. For example, there were a few examples of girls noting that their friends sometimes inhibited or discouraged them from being active.

Rebecca “Sometimes if we’re going to a certain place and we could walk and I’ll be like, ‘Oh, let’s walk!’, and they’re like, ‘Oh no, I don’t wanna walk. Let’s get the bus’.”

Similarly, in family situations girls noted that due to family members being busy with various separate physical activities sometimes they were too busy to spend time together.

## Discussion

This qualitative study explored adolescent girls’ perceptions of how social support influences their PA behaviour. Whilst it has been considered important to engage families and friends in PA interventions, there is limited research to guide effective intervention strategies to increase perceptions of social support. This study explored participants’ perspectives about how significant others support their PA participation and a grounded theory has been proposed.

Overall, a number of mechanisms influenced different domains of PA behaviour. Social support increased different domains of PA through enhancing self-efficacy, performance improvements, enhancing motivation, enhancing enjoyment, and enabling PA. Modelling PA provided opportunities for organized sport and leisure activities and inspired girls’ to take up or continue with organized sports. Current relationships presented opportunities for girls to receive social support and participating in PA in some cases added to the girl’s social networks and created additional opportunities to receive social support.

Consistent with previous findings (e.g., Motl et al., 2007; Peterson et al., 2013; Trost et al., 2003), the current study found that social support influences girls’ self-efficacy in their abilities to overcome barriers to PA, which in turn influences their PA levels. We also found that social support influenced girls’ task self-efficacy, or confidence in their abilities to be physically active. These findings suggest that social support is an important contributor of girls’ self-efficacy for PA, which has been consistently associated with girls’ PA levels (Biddle, Whitehead, O’Donovan, & Nevill, 2005; Craggs, Corder, van Sluijs, & Griffin, 2011). Increased self-efficacy from social support was linked to increases in motivation to be physically active. This mechanism suggests that the role of social support may partly be to enhance self-efficacy, which in turn could influence motivation for PA. This is consistent with evidence from previous studies and systematic reviews (Craggs et al., 2011; Sheridan, Coffee, & Lavallee, 2014), and psychological theories of behaviour that incorporate self-efficacy such as Social Cognitive Theory (Bandura, 2004). Building on this previous research, the ways in which social support contributed to increased self-efficacy were outlined in this study. Through positive reinforcement, social support contributed to increased task self-efficacy, and through providing a distraction from feeling insecure about being active alone and through overcoming lethargy social support contributed to increased barrier self-efficacy. These findings could provide a basis for intervention design, by outlining ways in which social support can influence self-efficacy. For example, positive reinforcement could be used as an intervention strategy to improve engagement in organized sports or PE.

The girls also reported that social support contributed to their enjoyment of physical activities, and this was evident across multiple domains of PA. Enjoyment was enhanced through coaches and teachers creating positive environments in which to be physically active (PE and organized sport), and through participating in physical activities with friends (PE, organized sport, active travel, and leisure activities). Together these findings suggest that social support can contribute to young people having fun, positive experiences, which contributes to their sustained engagement in PA. Whilst prior research has highlighted the importance of enjoyment in PA interventions (Camacho-Miñano, LaVoi, & Barr-Anderson, 2011; Loman, 2008), findings from the current study indicate that social support and involving friends or family may be particularly important for facilitating enjoyment of physical activities amongst adolescent girls.

The findings also suggest that social support can contribute to performance improvements and lead to sustained engagement in organized sports. The girls highlighted how performance improvements were motivating, which also contributed to their sustained engagement in sport. This study found that social support helped girls identify mistakes in their performance, provided them with knowledge of areas to improve, presented opportunities for them to practice and presented a broader range of activities to participate in. Considering this, identifying ways to help significant others to provide support that can lead to improvements in performance, for example providing parents with introductory coaching training in hockey, may be a successful intervention strategy for girls’ sustained engagement in organized sport.

The participants’ highlighted that instrumental forms of support enabled them to be physically active, for example through parents driving the girls to training practices, or paying for sports equipment and memberships. Not surprisingly, this was particularly evident for organized sports and leisure activities where logistic and financial support are needed. Intervention strategies that help significant others to identify logistic and other barriers to girls’ participation in PA, as well as possible ways of overcoming these barriers (e.g., knowledge of free or low cost sports or leisure activities available locally), may enable girls’ to engage in PA.

Significant others who modelled physical activities (e.g., participated in PA, or valued/were interested in PA) were able to provide girls with specific forms of support such as advice or instruction. Conversely, those who did not model PA lacked the knowledge or ability to provide more technical forms of support like instruction and also limited opportunities to engage in physical activities together. The findings of the study suggested that modelling was influential in two ways: by inspiring the girls, and by presenting opportunities for girls to be physically active.

Previous research has identified links between parent modelling and adolescent girls PA (Laird et al., 2016). Furthermore, links between parent enjoyment of PA and perceived importance of child PA (Dowda et al., 2011) and the levels of social support provided to children have been identified. This provides some indication that parents who enjoy PA and value the importance of child PA may be more likely to provide their child support than parents who do not enjoy or value PA for their child. This provides further support for targeting significant others in PA interventions aimed at adolescent girls.

Two potential mechanisms to explain friend modelling have previously been proposed: the peer contagion model (whereby young people are influenced by their friends’ activity levels) and the peer selection model (whereby individuals seek out friends with activity levels similar to their own) (Sawka et al., 2013). The current study found that modelling can inspire girls and present opportunities for them to be active, supporting the peer contagion model. However, girls may also seek out or become friends with those with activity levels or PA interests similar to their own. This was also evident in our findings, as girls discussed making friends as a result of their involvement in physical activities. These findings have implications for future research and practice. In particular, if provision of social support is related to modelling of PA it may also be necessary to target the PA behaviour of family and friends when attempting to increase adolescent girls’ PA. Targeting family and friends’ PA behaviour in interventions could enhance their capacity to provide support, whilst inspiring and offering more opportunities for young people to be physically active. There is some evidence of the effectiveness of this approach in the literature. For example, a recent review by Brown and colleagues (2016) found evidence for family-based interventions that used the child as the “agent of change” to model PA behaviour to their parents. Given that relatively few PA interventions have used “agent of change” or other modelling approaches, further interventions testing the efficacy of such approaches are warranted.

Finally, there appeared to be a link between participation in PA and connectedness, which was considered as the number and strength of social relationships. Girls discussed making friendships as a result of taking part in organized physical activities, how having shared interests in PA strengthened relationships, and how coaches and teachers were added to girls’ networks as a result of their participation in activities. New network members as a result of friendships from taking part in organized activities offered participants access to additional social support. However, in some cases, connectedness was negatively affected as a result of participating in physical activities where involvement in activities limited time spent with friends or family outside of that activity. This suggests there may be a bidirectional relationship between social support, PA and connectedness. Whilst having several and/or strong social connections has the potential to increase access to social support and hence PA, in some cases participating in PA could weaken social connections whereby friends or family members did not share an interest in PA. Given the importance of social networks of providing support and modelling PA and the link between PA and connectedness, intervention strategies could focus on building networks around PA presenting girls with access to social support and modelling. Such networks may protect girls during transitional or difficult phases when declines in PA are evident.

This study adds to previous research by providing an in-depth analysis of how social support influences PA in adolescent girls. This grounded theory adds to previous theoretical conceptualizations of social support by outlining how social support influences PA behaviour, which has not previously been fully explored. This grounded theory goes beyond prior research that has explored one or two prior mechanisms (e.g., through enjoyment and self-efficacy), by suggesting five mechanisms through which social support can influence PA, and it also highlights the importance of understanding the role of modelling and social networks. In addition, as demonstrated in Figure 1, this study links the mechanisms through which social support influences PA behaviour to different domains of PA. This provides a clear starting point for developing interventions aimed at increasing different domains of PA in adolescent girls through provision of social support. Whilst all of the mechanisms outlined in Figure 1 have potential to inform the design of intervention strategies, targeting self-efficacy, performance, enjoyment, motivation and providing opportunities for PA, may be the most feasible given that providing financial support or modelling successes in PA could be more challenging. However, developmental work is needed to design and pilot such intervention strategies.

Limitations to the study should also be considered in relation to the findings. This study purposely targeted girls who were active and the findings reflect different domains of PA, however, the majority of the participants were involved in organized sports. Whilst previous research has highlighted that being involved in organized sport is a strong determinant of PA levels in adolescent girls (Biddle et al., 2005), it is possible that girls who are not involved in organized sport may have different responses to social support than inactive girls. Intervention studies are therefore warranted to test potential social support intervention strategies identified in this study. This study also purposely targeted only adolescent girls to consider how *perceived* social support influences PA behaviour. It is also important to note that whilst *received* social support appears to have weaker or inconsistent associations with health outcomes (Uchino et al., 2012), understanding providers’ perspectives of the ways in which the social support that they provide influences adolescent girls PA behaviour may add to the grounded theory. These limitations, in addition to the small sample size recruited from an urban area in the UK, may have implications on the generalizability of the findings to girls in other settings or contexts. It is also unclear if the results can be applied to developing intervention strategies for inactive girls, and further research should explore development of such strategies.

These findings have implications for (1) further research exploring the role of social support on PA behaviour, and (2) PA intervention design. Adding to and refining this grounded theory will further improve understanding on how social support influences PA in adolescent girls. There are also implications for PA intervention design for adolescent girls. PA interventions could target the mechanisms outlined in Figure 1 to target-specific domains of PA and use the information under each mechanism in Figure 1 to guide intervention design. Testing these mechanisms in PA interventions could also further refine the grounded theory and our understanding of how social support influences PA.

## Conclusion

Using a constructivist grounded theory approach, this study has comprehensively investigated how adolescent girls perceive social support to influence their PA behaviour. A grounded theory of adolescent girls’ perceptions and experiences of how social support influences PA was developed. The grounded theory outlines potential mechanisms through which social support influences different domains of PA in adolescent girls and provides a framework for future research examining the role of social support in PA. As grounded theories are modifiable, as previously outlined, research could further develop this grounded theory or expand its application to other populations. The grounded theory could also be used to inform PA intervention design, specifically, through informing development of strategies to increase girls’ perceptions of social support across different domains of PA.

## Geolocation information section

Scotland, UK.

## References

[CIT0001] Bandura A. (2004). Health promotion by social cognitive means. *Health Education & Behavior*, 31(2), 143–14. 15090118 10.1177/1090198104263660

[CIT0002] Beets M. W., Cardinal B. J., & Alderman B. L. (2010). Parental social support and the physical activity-related behaviors of youth: A review. *Health Education and Behavior*, 37(5), 621–644. 20729347 10.1177/1090198110363884

[CIT0003] Biddle S. J., Whitehead S., O’Donovan T., & Nevill M. (2005). Correlates of participation in physical activity for adolescent girls: A systematic review of recent literature. *Journal of Physical Activity and Health*, 2, 423–434.

[CIT0004] Biddle S. J. H., Atkin A. J., Cavill N., & Foster C. (2011). Correlates of physical activity in youth: A review of quantitative systematic reviews. *International Review of Sport and Exercise Psychology*, 4(1), 25–49.

[CIT0005] Brown H. E., Atkin A. J., Panter J, Wong G., Chinapaw M. J., & Sluijs E. M. F. (2016). Family-based interventions to increase physical activity in children: a systematic review, meta-analysis and realist synthesis. Reviews, 17(4), 345–360. doi:10.1111/obr.12362 PMC4819691 26756281

[CIT0006] Camacho-Miñano M. J., LaVoi N. M., & Barr-Anderson D. J. (2011). Interventions to promote physical activity among young and adolescent girls: A systematic review. *Health Education Research*, 26(6), 1025–1049. 21680763 10.1093/her/cyr040

[CIT0007] Caspersen C. J., Powell K. E., & Christenson G. M. (1985). Physical activity, exercise, and physical fitness: Definitions and distinctions for health-related research. *Public Health Reports*, 100(2), 126. 3920711 PMC1424733

[CIT0008] Charmaz K. (2014). *Constructing grounded theory* (2nd ed.). London, UK: SAGE Publications.

[CIT0009] Charmaz K. (2006). Grounded theory In Ritzer G. (Ed.), *Encyclopedia of sociology*. Cambridge, MA: Blackwell.

[CIT0010] Craggs C., Corder K., van Sluijs E. M. F., & Griffin S. J. (2011). Determinants of change in physical activity in children and adolescents: A systematic review. *American Journal of Preventive Medicine*, 40(6), 645–658. 21565658 10.1016/j.amepre.2011.02.025 PMC3100507

[CIT0011] Currie C., Van der Sluijs W., Whitehead R., Currie D., Rhodes G., Neville F., & Inchley J. (2015). *Health behaviour in school-aged children (HBSC) 2014 survey in Scotland National Report*. Scotland, UK: Child and Adolescent Health Research Unit (CAHRU). Retrieved from http://www.cahru.org/content/03-publications/04-reports/hbsc_nr14_interactive_final.pdf.

[CIT0012] Department of Health. (2011). *Physical activity guidelines for children and young people (5–18 years).*London, UK: Department of Health.

[CIT0013] Ding D., Lawson K. D., Kolbe-Alexander T. L., Finkelstein E. A., Katzmarzyk P. T., van Mechelen W., & Pratt M. (2016). The economic burden of physical inactivity: A global analysis of major non-communicable diseases. *The Lancet*, 388(10051), 1311–1324. 10.1016/S0140-6736(16)30383-X 27475266

[CIT0014] Dowda M., Pfeiffer K. A., Brown W. H., Mitchell J. A., Byun W., & Pate R. R (2011). Parental and environmental correlates of physical activity of children attending preschool.archives. Medicine, 165(10), 939–944. 10.1001/archpediatrics.2011.84 21646573

[CIT0015] Dunton G. F., Schneider M., & Cooper D. M. (2007). An investigation of psychosocial factors related to changes in physical activity and fitness among female adolescents. *Psychology & Health*, 22(8), 929–944.

[CIT0016] Edwardson C. L., & Gorely T. (2010). Parental influences on different types and intensities of physical activity in youth: A systematic review. *Psychology of Sport and Exercise*, 11(6), 522–535.

[CIT0017] Elo S., Kääriäinen M., Kanste O., Pölkki T., Utriainen K., & Kyngäs H. (2014). Qualitative content analysis. *A Focus on Trustworthiness*, 4(1). doi:10.1177/2158244014522633

[CIT0018] Glaser B. G., & Straus A. L. (1967). *The discovery of grounded theory*. Chicago: Aldine.

[CIT0019] Hallal P. C., Andersen L. B., Bull F. C., Guthold R., Haskell W., & Ekelund U.; Lancet Physical Activity Series Working, G (2012). Global physical activity levels: Surveillance progress, pitfalls, and prospects. *Lancet*, 380(9838), 247–257. 22818937 10.1016/S0140-6736(12)60646-1

[CIT0020] Heaney C. A., & Israel B. A. (2008). Social networks and social support In Glanz K., Rimer B. K., & Viswanath K. (Eds.), *Health behavior and health education: Theory, research and practice* (4th ed.). San Francisco, CA: Jossey-Bass.

[CIT0021] Holt-Lunstad J., Smith T. B., & Layton J. B. (2010). Social relationships and mortality risk: A meta-analytic review. *PLoS Medicine*, 7(7), e1000316. 20668659 10.1371/journal.pmed.1000316 PMC2910600

[CIT0022] Jago R., Brockman R., Fox K. R., Cartwright K., Page A. S., & Thompson J. L (2009). Friendship groups and physical activity: qualitative findings on how physical activity is initiated and maintained among 10–11 year old children. Activity, 6(1), 4. doi:10.1186/1479-5868-6-4 PMC2631002 19138411

[CIT0023] Janssen I., & LeBlanc A. G. (2010). Systematic review of the health benefits of physical activity and fitness in school-aged children and youth. *International Journal of Behavioral Nutrition and Physical Activity*, 7, 40. 20459784 10.1186/1479-5868-7-40 PMC2885312

[CIT0024] Laird Y., Fawkner S., Kelly P., McNamee L., & Niven A. (2016). The role of social support on physical activity behaviour in adolescent girls: A systematic review and meta-analysis. *International Journal of Behavioral Nutrition and Physical Activity*, 13(1), 1–14. 27387328 10.1186/s12966-016-0405-7 PMC4937604

[CIT0025] Langford C. P. H., Bowsher J., Maloney J. P., & Lillis P. P. (1997). Social support: A conceptual analysis. *Journal of Advanced Nursing*, 25, 95–100. 9004016 10.1046/j.1365-2648.1997.1997025095.x

[CIT0026] Loman D. G. (2008). Promoting physical activity in teen girls: Insight from focus groups. *MCN. The American Journal of Maternal Child Nursing*, 33(5), 294–299; quiz 300–301. 18758332 10.1097/01.NMC.0000334896.91720.86

[CIT0027] Lubans D. R., & Sylva K. (2009). Mediators of change following a senior school physical activity intervention. *Journal of Science and Medicine in Sport*, 12(1), 134–140. 18069061 10.1016/j.jsams.2007.08.013

[CIT0028] McAuley E., & Mihalko S. L. (1998). Measuring exercise-related self-efficacy In *Advances in sport and exercise psychology measurement* (pp. 371–390). Morgantown, WV: Fitness Information Technology.

[CIT0029] McDowell T. L., & Serovich J. M. (2007). The effect of perceived and actual social support on the mental health of HIV-positive persons. *AIDS Care*, 19(10), 1223–1229. 18071966 10.1080/09540120701402830 PMC2151198

[CIT0030] Mitchell J. C. (1969). *The concept and use of social networks*. Manchester, UK: Manchester University Press.

[CIT0031] Motl R. W., Dishman R. K., Saunders R. P., Dowda M., & Pate R. R. (2007). Perceptions of physical and social environment variables and self-efficacy as correlates of self-reported physical activity among adolescent girls. *Journal of Pediatric Psychology*, 32(1), 6–12. 16707779 10.1093/jpepsy/jsl001

[CIT0032] O’Reilly M., & Parker N. (2012). ‘Unsatisfactory Saturation’: A critical exploration of the notion of saturated sample sizes in qualitative research. *Qualitative Research*, 13(2), 190–197.

[CIT0033] Pearson N., Braithwaite R., & Biddle S. J. (2015). The effectiveness of interventions to increase physical activity among adolescent girls: A meta-analysis. *Academic Pediatrics*, 15(1), 9–18. 25441655 10.1016/j.acap.2014.08.009

[CIT0034] Peterson M., Lawman H., Wilson D., Fairchild A., & Van Horn M. (2013). The association of self-efficacy and parent social support on physical activity in male and female adolescents. *Health Psychology*, 32(6), 666–674. 22888813 10.1037/a0029129 PMC3502660

[CIT0035] Poitras V. J., Gray C. E., Borghese M. M., Carson V., Chaput J.-P., Janssen I., … Tremblay M. S. (2016). Systematic review of the relationships between objectively measured physical activity and health indicators in school-aged children and youth. *Applied Physiology, Nutrition, and Metabolism*, 41(6 (Suppl. 3)), S197–S239. 10.1139/apnm-2015-0663 27306431

[CIT0036] Prochaska J. J., Sallis J. F., & Long B. (2001). A physical activity screening measure for use with adolescents in primary care. *Archives of Pediatrics & Adolescent Medicine*, 155(5), 554–559. 11343497 10.1001/archpedi.155.5.554

[CIT0037] Sabiston C. M., McDonough M. H., & Crocker P. R. (2007). Psychosocial experiences of breast cancer survivors involved in a dragon boat program: Exploring links to positive psychological growth. *Journal of Sport and Exercise Psychology*, 29(4), 419–438. 17968046 10.1123/jsep.29.4.419

[CIT0038] Sallis J. F., Owen N., & Fotheringham M. J. (2000). Behavioral epidemiology: A systematic framework to classify phases of research on health promotion and disease prevention. *Annals of Behavioral Medicine*, 22(4), 294–298. 11253440 10.1007/BF02895665

[CIT0039] Sawka K. J., McCormack G. R., Nettel-Aguirre A., Hawe P., & Doyle-Baker P. K (2013). Friendship networks and physical activity and sedentary behavior among youth: a systematized review. *International Journal of Behavioral Nutrition and Physical Activity*, 10(1), 130. doi:10.1186/1479-5868-10-130 24289113 PMC4220781

[CIT0040] Shen B., Centeio E., Garn A., Martin J., Kulik N., Somers C., & McCaughtry N. (2016). Parental social support, perceived competence and enjoyment in school physical activity. *Journal of Sport and Health Science*. doi:10.1016/j.jshs.2016.01.003 PMC6189254 30356633

[CIT0041] Sheridan C. L., & Radmacher S. A. (1992). *Health psychology: Challenging the biomedical model*. Oxford, UK: John Wiley & Sons.

[CIT0042] Sheridan D., Coffee P., & Lavallee D. (2014). A systematic review of social support in youth sport. *International Review of Sport and Exercise Psychology*, 7(1), 198–228.

[CIT0043] Standiford A. (2013). The secret struggle of the active girl: A qualitative synthesis of interpersonal factors that influence physical activity in adolescent girls. *Health Care for Women International*, 34(10), 860–877. 23790150 10.1080/07399332.2013.794464

[CIT0044] Telford R. M., Telford R. D., Olive L. S., Cochrane T., & Davey R. (2016). Why are girls less physically active than boys? Findings from the LOOK longitudinal study. *PLoS One*, 11(3), e0150041. 26960199 10.1371/journal.pone.0150041 PMC4784873

[CIT0045] The Physical Activity for Health Alliance (2010). *Five-year review of let’s make Scotland more active: A strategy for physical activity*. Edinburgh: NHS Health Scotland.

[CIT0046] Trost S. G., Sallis J. F., Pate R. R., Freedson P. S., Taylor W. C., & Dowda M. (2003). Evaluating a model of parental influence on youth physical activity. *American Journal of Preventive Medicine*, 25(4), 277–282. 14580627 10.1016/s0749-3797(03)00217-4

[CIT0047] Uchino B. N., Bowen K., Carlisle M., & Birmingham W. (2012). Psychological pathways linking social support to health outcomes: A visit with the “ghosts” of research past, present, and future. *Social Science & Medicine*, 74(7), 949–957. 22326104 10.1016/j.socscimed.2011.11.023 PMC3298603

[CIT0048] van Stralen M. M., Yildirim M., Velde S. J. T., Brug J., van Mechelen W., & Chinapaw M. J. M. (2011). What works in school-based energy balance behaviour interventions and what does not? A systematic review of mediating mechanisms. *International Journal of Obesity*, 35(10), 1251–1265. 21487398 10.1038/ijo.2011.68 PMC3191379

[CIT0049] Verloigne M., Veitch J., Carver A., Salmon J., Cardon G., De Bourdeaudhuij I., & Timperio A. (2014). Exploring associations between parental and peer variables, personal variables and physical activity among adolescents: A mediation analysis. *BMC Public Health*, 14, 966. 25231062 10.1186/1471-2458-14-966 PMC4175273

[CIT0050] Weed M. (2009). Research quality considerations for grounded theory research in sport & exercise psychology. *Psychology of Sport and Exercise*, 10(5), 502–510.

[CIT0051] Weiss M. R., Smith A. L., & Theeboom M. (1996). “That’s what friends are for”: Children’s and teenagers’ perceptions of peer relationships in the sport domain. *Journal of Sport & Exercise Psychology*, 18(4), 347–379.

[CIT0052] Wing E. K., Bélanger M., & Brunet J. (2016). Linking parental influences and youth participation in physical activity in- and out-of-school: The mediating role of self-efficacy and enjoyment. *American Journal of Health Behavior*, 40(1), 31–37. 26685811 10.5993/AJHB.40.1.4

[CIT0053] Yao C. A., & Rhodes R. E. (2015). Parental correlates in child and adolescent physical activity: A meta-analysis. *International Journal of Behavioral Nutrition and Physical Activity*, 12(1), 10. 10.1186/s12966-015-0163-y PMC4363182 25890040

